# Latest Review Papers in Molecular Plant Sciences 2023

**DOI:** 10.3390/ijms25105407

**Published:** 2024-05-15

**Authors:** Setsuko Komatsu, Andrei Smertenko

**Affiliations:** 1 Faculty of Environmental and Information Sciences, Fukui University of Technology, Fukui 910-0028, Japan; 2 Institute of Biological Chemistry, Washington State University, Washington, WA 99164-7411, USA

Success in sustaining food security in the face of global climate change depends on the multi-disciplinary efforts of plant science, physics, mathematics, and computer sciences, whereby each discipline contributes specific concepts, information, and tools. Rapidly accumulating volumes of experimental data about diverse aspects of plant life challenge existing approaches aiming at the interrogation of data and using it to construct models that can predict interactions between genotype and environment. The key role of molecular plant sciences remains to collect reliable information about the fundamental processes of plant biology and organize it in formats that can be used by scientists working in other disciplines. This Special Issue, titled “Latest Review Papers in Molecular Plant Sciences 2023”, aims to collect review papers in all fields of plant sciences. In particular, the aim is to illustrate frontier research in molecular plant science through six review articles covering several important advances in diverse topics. Two articles summarized plant responses to abiotic stress [1,2], two articles addressed responses to biotic stress [3,4], and two articles focused on methodology [5,6]. 

The erratic weather patterns impose multiple stresses on plants, including a higher content of salts in the soil (salinity), reduced soil moisture content (drought), extremely high day and night temperatures, non-seasonal temperature decreases, and floods [7]. Another consequence of climate change is the spreading of plant pathogens to new geographical locations where previously the weather conditions were suboptimal for the disease. Collectively, the stressful environmental conditions and pathogens can lead to partial or complete yield losses in the affected areas [8]. The yield losses impact food availability, resulting in socio-economic insecurities along with health implications, particularly in marginalized populations [9,10]. Considering the current rate of population growth, sustaining food security requires increasing agricultural output by as much as 50–70% [11]. One of the key contributions of plant molecular biology for sustaining food security focuses on comprehending the impact of abiotic and biotic stress on plants and mechanisms of resiliency to environmental changes.

The introduction of traits associated with greater environmental resiliency into commercial crop varieties using a combination of genomic selection and classical breeding techniques offers an effective strategy for sustaining yields. However, the specificity and sensitivity of phenotyping approaches remain the major bottlenecks for the identification of relevant genetic markers. Advances in omics technologies, including genomics, transcriptomics, proteomics, and metabolomics, paved the way for accelerating the discovery of genetic markers using molecular phenotyping [12,13,14]. Combining multiple omic approaches to a specific experimental condition in the so-called “muti-omics” studies contributes to comprehending the plasticity of metabolic pathways and determining the functions of regulatory genetic networks that control metabolic processes under various environmental conditions in diverse plant species [14,15]. 

Next-generation sequencing and advanced mass spectrometry enable higher throughput and faster data collection in the multi-omics experiments [16,17], leading to greater accuracy and sensitivity in identifying genetic markers associated with stress resiliency [18]. A complementary technique, organellomics, offers quantitative data on cellular responses under different environmental conditions, hence leading to a deeper comprehension of changes at the molecular level [19]. For example, peroxisome abundance increases in response to heat and drought stress in wheat and quinoa, and peroxisome abundance negatively correlates with yield [20,21]. Pathogens exploit cellular pathways to overcome plant immunity as successful infection with the Wheat Stripe Mosaic Virus was shown to be accompanied by lower peroxisome abundance [22]. Integrating organellomics data and data generated by other techniques with whole-plant phenotyping becomes critical for developing predictive models in plant stress physiology.

Among the most common abiotic stresses, soil salinity is one of the key factors contributing to the decline in crop yields worldwide [23]. The article by Kausar and Komatsu [1] provides an overarching review of morphological, physiological, and molecular responses to salinity stress and relevant tolerance mechanisms in crops. The significance of proteomic approaches for improving salt tolerance of various crops is highlighted, and the contributions of integrated omics approaches to achieving global food security are discussed in the context of previous research findings [24]. Proteomics refers to the study of protein properties, including abundance, post-translational modifications, and protein-protein interactions [25]. For example, proteins related to the reactive-oxygen species scavenging system and abscisic-acid activation are found exclusively in salt-tolerant rice varieties that are capable of accumulating the auxin [26]. The authors propose that novel proteins showing a response to salinity and a correlation with salt stress tolerance are potential candidates for breeding resilient crops.

Another important topic covered in this Special Issue is the relationship between stress and flowering. Regulation of flowering time relies on sensing seasonal changes in the photoperiod [27]. The CONSTANS-like (COL) protein family belongs to the group of photoperiod-sensitive transcription factors with a crucial role in flowering [28]. The article by Zhang et al. [2] highlights recent advances in the characterization of the structure of the COL proteins and their regulatory patterns within transcription complexes. Novel findings about the dual role of COL proteins in flowering and abiotic stress responses provide valuable references for untangling the contribution of COL to stress resilience and exploring the relationship between reproduction and environmental cues.

A major pest of rice, the brown planthopper (BPH), causes significant yield losses in rice [29] by damaging above-ground tissues during the feeding process with piercing-sucking mouthparts. This pathogen is commonly controlled using chemical insecticides [30]. As insecticides affect wildlife, genetic resistance mechanisms are of primary importance. The review by Yan et al. [3] summarized advances in molecular mechanisms for BPH pathogenicity and the breeding of BPH-resistant rice varieties. Genetic analysis led to the identification of 70 genes and a quantitative trait locus (QTL) associated with BPH resistance in rice. However, efficient utilization of these markers required gene/QTL duplication in as many as 26 known cases [31,32,33,34]. The insights from this review could advance the development of sustainable biological control strategies for managing BPH infestations.

*L. plantarum* is one of the most ubiquitous lactic acid bacteria isolated all over the world from spontaneously fermented plant material [35]. This environmental adaptability highlights the great ecological, technological, and therefore scientific and economic importance of *L. plantarum* [36]. Skotniczny and Satora [4] provided an overview of molecular techniques, both culture-dependent and culture-independent, currently used to detect and identify *L. plantarum*. Due to the close relationship between the lactic acid bacteria and *L. plantarum* species, traditional methods for detection and identification of these species may yield erroneous data. They concluded that quantitative polymerase chain reaction with culture-independent methods provides the most reliable and practical approach for the identification of *L. plantarum,* whereas commonly used 16S rRNA and shotgun sequencing using next- and third-generation sequencers work better for characterizing the composition of microbial communities than for the identification of single species.

Determining gene functions using genome editing technologies offers a simpler experimental design, higher efficiency, and lower costs in comparison with reverse genetics approaches [37]. However, strong developmental phenotypes or lethality associated with permanent genetic modifications can confound the function and characterization of the genes of interest. More recently, it was reported that fusing the clustered regularly interspaced short palindromic repeats (CRISPR) associated 9 (Cas9) protein with methylation- and histone-modifying enzymes can be used for targeted modulation of the target gene expression by epigenetic mechanisms [38]. Qi et al. [5] summarized the recent progress in epigenome editing by CRISPR-Cas9-related approaches and outlined directions for future development of this technique in plants. They provided examples of how a combination of targeted epigenetic changes and the rapid development of relevant gene editing techniques could be applied to improve important traits in crops.

Endoreplication represents a common modification of the cell cycle in both plants [39] and animals [40] that involves genome replication without subsequent cell division. The final outcome of endoreplication is endopolyploidy. The article by Kołodziejczyk et al. [6] describes the potential of this phenomenon for plant biotechnology. In particular, endopolyploidy can increase the expression of metabolic and stress-tolerance genes [41]. Exploiting the endoreplication in biotechnology and agriculture requires comprehension of approaches for effective and precise induction and sustaining of this process.

Progress in current plant research offers exciting opportunities for advancing tolerance to both biotic and environmental factors by optimizing the expression of functional genes in plants under climate change [42,43,44]. With the rapid innovations in high-throughput technologies, plant stress research begins to tackle the phenomenon of stress memory. The stress memory research is expected to progress from a single-omics level to multi-omics cross-correlation analyses to the systematic construction of the underlying genetic regulatory networks on the scales of molecules, cells, whole plants, and populations. The Editors believe that the review articles published in this Special Issue provide useful information for fundamental research, the development of new applications in plant breeding, and entrepreneurship.
